# Early functional outcomes after condylar-stabilizing (deep-dish) versus standard bearing surface for cruciate-retaining total knee arthroplasty

**DOI:** 10.1186/s43019-019-0001-7

**Published:** 2019-06-28

**Authors:** P. Stirling, N. D. Clement, D. MacDonald, J. T. Patton, R. Burnett, G. J. Macpherson

**Affiliations:** Department of Orthopaedics and Trauma, The Royal Infirmary of Edinburgh, Little France, Edinburgh, EH16 4SA UK

**Keywords:** Total knee arthroplasty, Outcome, Bearing surface, Deep dish, Cruciate retaining

## Abstract

**Aims:**

The primary study aim was to compare early knee-specific function of patients undergoing cemented total knee arthroplasty (TKA) with either a cruciate-retaining (CR) polyethylene insert or a highly congruent condylar-stabilizing (CS) insert. Secondary aims were to compare general health and satisfaction between the groups.

**Methods:**

A total of 418 consecutive primary TKAs were identified retrospectively. Demographics and preoperative and 1-year postoperative patient-reported outcome measures (PROMs) were collected prospectively. PROMs consisted of Oxford Knee Scores, EuroQol-5 Dimensions scores, and Short Form-12 scores.

**Results:**

A total of 54 (12.9%) patients received a CS insert and 364 patients received a CR TKA. The CS group had a significantly (odds ratio (OR) 2.9; *p* = 0.002) greater proportion of females (77.8% versus 54.9%). The only significant difference in postoperative PROMs was a higher Short Form-12 physical component score in the CR group (difference 3.1; 95% confidence interval (CI) 0.1 to 6.1; *p* = 0.04). Linear regression analysis demonstrated no significant difference for all postoperative PROMs (*p* > 0.25). There was no significant difference in satisfaction rate (OR 0.94; 95% CI 0.42 to 2.12; *p* = 0.56) or pain visual analogue score (difference 6.1; 95% CI –1.9 to 14.0; *p* = 0.14) between the groups.

**Conclusion:**

More congruent CS inserts have equivalent PROMs and patient satisfaction at 1 year compared with less congruent CR inserts. These represent an option for surgeons undertaking TKA where increased congruency is desired.

## Introduction

Improving the range of motion while preserving joint stability in the coronal and sagittal planes are fundamental objectives of primary total knee arthroplasty (TKA). There is no consensus regarding preservation or removal of the posterior cruciate ligament (PCL) in primary TKA. Depending on the surgeon’s preference, the PCL can be sacrificed and a posterior-stabilizing (PS) implant with a cam can be used or the PCL can be preserved with use of a cruciate-retaining (CR) prosthesis. Concerns have been raised over the functionality of a preserved PCL, with a cadaveric study revealing normal PCL strain in only 37% of CR TKAs [1]. This concern must be balanced against the potential disadvantages associated with PS systems including patellar clunk syndrome [2], increased polyethylene wear [3], and additional femoral bone resection.

**Fig. 1:**
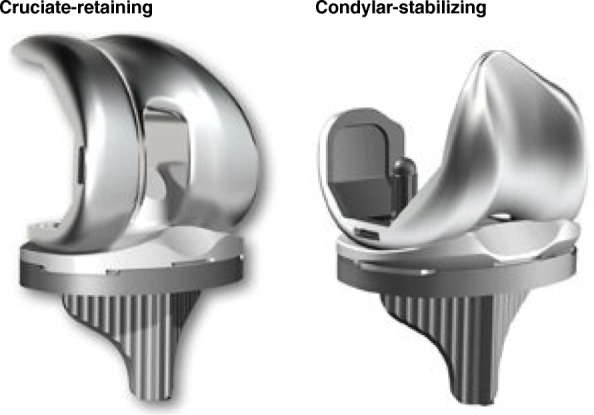
Structural differences between condylar-stabilizing and cruciate-retaining total knee replacement

Surgeons using a CR prosthesis must carefully assess the PCL intraoperatively, and if it is found to be absent or incompetent, then an increased level of constraint may be achieved by use of a PS implant. Additional congruency may also be desired in the presence of a PCL which is present but attenuated or in the case of a flexion/extension mismatch. CR femoral and tibial components can still be used in this scenario by introducing additional congruency with an anterior-lipped condylar-stabilizing (CS) tibial bearing surface. Structural differences between CR and CS tibial inserts are illustrated in Fig. 1. Berend et al. [4] compared the range of movement (ROM) of patients who had a CR prosthesis with either a standard bearing surface or a deep-dish highly congruent bearing surface for a deficient PCL. They demonstrated a greater improvement in ROM at 6 weeks for the group with a deep dish with no early revisions for instability. What remains unknown is whether early patient-reported outcome measures (PROMs) and satisfaction are influenced by the use of a standard bearing surface when the PCL is competent compared with a deep-dish highly congruent CS bearing surface when the PCL is present but attenuated.

The primary aim of this study was to compare early knee-specific function of patients with a standard bearing surface when the PCL is competent with those who had a CS bearing surface when the PCL was attenuated. The secondary aims were to compare general health and patient satisfaction between the groups. The null hypothesis is that there is no difference in early knee-specific functional outcome between the groups.

## Methods

Ethical approval was obtained from the regional ethics committee (Research Ethics Committee, South East Scotland Research Ethics Service, Scotland, 11/AL/0079) for collection, analysis, and publication of the anonymised data collected for this study.

During a 1-year period (2013), patients undergoing the same CR TKA at the study centre had outcome data recorded prospectively. The study cohort was retrospectively identified: the bearing surface used was recorded from theatre implant logbooks held at the study centre. The inclusion criterion for this study was a primary TKA with no extra-articular deformity, with available outcome data recorded preoperatively and 1 year postoperatively. Exclusion criteria were revision for infection in the first year, patients undergoing simultaneous bilateral TKAs, and patients without available postoperative data. Patients undergoing consecutive bilateral TKAs during the study period only had outcome measures assessed for their first knee (*n* = 6).

The patient demographics, comorbidities, body mass index (BMI), and patient-reported outcome measures were recorded at the preoperative assessment clinic. The Oxford Knee Score (OKS) [5] and the Short Form-12 (SF-12) score [6] were recorded preoperatively and at 1 year postoperatively via postal questionnaire. The OKS consists of 12 questions assessed on a Likert scale with values from 0 to 4. A summative score is then calculated where 48 is the best possible score (least symptomatic) and 0 is the worst possible score (most symptomatic). The EuroQol (EQ) general health questionnaire [7] evaluates five domains (EQ-5D), which include mobility, self-care, usual activities, pain/discomfort, and anxiety/depression. This is then scored from less than 0 (a state worse than death) to 1.0, which represents a perfect health state. The SF-12 is a generic assessment tool to measure a patient’s well-being, which is assessed using a physical component summary (PCS) and a mental component summary (MCS). Both the SF-12 PCS and MCS range from 0% (worst level of functioning) to 100% (best level of functioning). Patients were asked to score their knee pain on a visual analogue scale (VAS) from 0 (worst pain) to 100 (no pain). The OKS and SF-12 were selected as these scores have been shown in a systematic review to be the best-performing knee-specific and generic outcome scores [8].

Patient satisfaction was assessed by asking the question “How satisfied are you with your operated knee?” 1 year after surgery. The response was recorded using a 4-point Likert scale: very satisfied, satisfied, uncertain, and unsatisfied. Patients who recorded very satisfied or satisfied were classified as satisfied.

During the study period, a consultant performed or scrub-supervised all included TKAs. Six consultant surgeons undertook all CS TKAs in this study. In addition to these six, an additional seven consultants performed CR TKAs during the study period which were included in the analysis. All patients underwent a cemented Triathlon (Stryker®, Kalamazoo, MI, USA) TKA using a measured resection technique. A mid-line medial parapatellar approach was made in all patients. Femoral component sizing and rotation were performed manually. The conventional jig alignment technique used intramedullary referencing for the femur and extramedullary for the tibia. The specified bone cuts were 5 degrees of valgus for the distal femoral cut and posterior condylar referencing for rotational alignment. The tibial bone cut was made to produce neutral varus/valgus alignment in the coronal plane with 3 degrees of posterior slope. The decision to use a CS insert was made following intraoperative assessment of the PCL. If the PCL was present and competent, a standard CR insert was used. If, however, the PCL was present but attenuated, or there were concerns regarding anteroposterior instability or a flexion/extension mismatch, a CS insert was utilized. All patients received three perioperative doses of prophylactic antibiotics (cefuroxime) and 4 weeks of postoperative pharmacological deep venous thrombosis (DVT) prophylaxis. A standardized rehabilitation protocol as per the local clinical care pathway was used for all patients, with active mobilization and full weight-bearing on the first day postoperatively. Patients were then reviewed at 6 weeks, 6 months, and 12 months postoperatively.

Statistical analysis was performed using Statistical Package for Social Sciences version 17.0 (SPSS Inc., Chicago, IL, USA). Parametric and non-parametric tests were used to assess continuous variables for significant differences between groups. A Student’s unpaired *t* test was used to compare linear variables between groups. Dichotomous variables were assessed using a chi-square test. Linear and binary logistic regression analysis was used to adjust for confounding variables between the groups to assess the independent effect of the bearing surface on the PROMs and patient satisfaction, respectively. *p* < 0.05 was defined as significant.

A post hoc power calculation was performed using the OKS (primary outcome measure), which has a defined minimal clinically important difference of 5 points [9] and a standard deviation (SD) of 10 points. This determined that 54 patients in the CS group and 364 patients in the control group achieved a power of 92.8% using two-tailed analysis and an α value of 0.05.

## Results

There were 418 TKAs performed during the study period that met the inclusion and exclusion criteria. A total of 54 patients (13%) underwent TKA with a highly congruent CS bearing surface and 364 patients received a standard CR insert. The mean age of the study cohort (CS and CR) was 68.7 years (range 42.7–89.7 years). There were 242 females (57.9%) and 176 males (42.1%) with a mean BMI of 31.3 kg/m^2^ (range 18.0–53.8 kg/m^2^). The most prevalent comorbidities in both groups were hypertension followed by rheumatoid arthritis and depression (Table 1). A one-way ANOVA revealed no significant differences in change in OKS between surgeons undertaking TKR with a CS insert (*p* = 0.13).

**Table 1:** Demographic case-mix variables according to group

Case-mix variable		CS (*n* = 54)	CR (*n* = 364)	*p* value
Age (years), mean (SD)		68.5 (9.4)	68.8 (8.6)	0.81*
Gender, *n* (% of group)	Male	12 (22.2)	164 (45.1)	0.002**
	Female	42 (77.8)	200 (54.9)	
BMI (kg/m^2^), mean (SD)		31.5 (8.1)	31.1 (5.6)	0.68*
Comorbidity, *n* (% of group)				
Diabetes	Yes	8 (14.0)	41 (11.0)	0.45**
	No	46 (86.0)	323 (89.0)	
Rheumatoid arthritis	Yes	10 (18.5)	52 (14.3)	0.41**
	No	44 (81.5)	312 (85.7)	
Back pain	Yes	25	156	0.63**
	No	29	208	
Depression	Yes	10	49	0.32**
	No	44	315	
Ischaemic heart disease	Yes	5 (9.3)	46 (12.6)	0.5**
	No	49 (90.7)	318 (87.4)	
Hypertension	Yes	25 (46.3)	182 (50.0)	0.8**
	No	29 (53.7)	182 (50.0)	

*t test

**Chi-square test

Bold numbers represent *p*-values of less than 0.1

*BMI* body mass index, *CR* cruciate retaining, *CS* condylar stabilizing, *SD* standard deviation

The CS group had a significantly greater proportion of female patients compared with the CR group (77.8% versus 54.9%; odds ratio (OR) 2.9; 95% confidence interval (CI) 1.5 to 5.6; *p* = 0.002). There were no other significant differences between the groups for age, BMI, or comorbidities (Table 1). There was a trend towards worse PROMs in the CS group (OKS, EQ-5D, and SF-12) preoperatively but there was no statistically significant difference identified (Table 2).

**Table 2:** Preoperative functional measures according to group

PROM	CS (*n* = 54)	CR (*n* = 364)	*p* value*
OKS, mean (SD)	19.9 (8.2)	21.0 (7.6)	0.3
EQ-5D, mean (SD)	0.41 (0.31)	0.45 (0.30)	0.44
SF-12 PCS, mean (SD)	31.0 (7.5)	32.8 (7.5)	0.1
SF-12 MCS, mean (SD)	47.3 (8.8)	47.7 (8.1)	0.74
Pain VAS, mean (SD)	52.9 (21.4)	51.8(20.5)	0.73

*t test

*CR* cruciate retaining, *CS* condylar stabilizing, *EQ-5D* EuroQol-5 Dimensions score, *OKS* Oxford Knee Score, *PROM* Patient-reported functional outcome measure, *SD* standard deviation, *SF-12 MCS* Short Form-12 mental component summary score, *SF-12 PCS* Short Form-12 physical component summary score, *VAS* visual analogue scale

The postoperative SF-12 physical component score was significantly higher in the CR group compared with the CS group (difference 3.1; 95% CI 0.1 to 6.1; *p* = 0.04). Higher postoperative scores were observed in the CR group but these differences were not found to be significant (Table 3). The mean improvement in OKS was equal in both groups (CS 13.5 ± 9.6 versus CR 13.6 ± 9.1; difference 0.1; 95% CI − 2.5 to 2.7; *p* = 0.94). Linear regression analysis demonstrated no significant difference between the groups for any of the PROMs assessed when adjusting for confounding variables (Table 4).

**Table 3:** Postoperative functional measures according to group

PROM	CS (*n* = 54)	CR (*n* = 364)	*p* value*
OKS, mean (SD)	33.2 (9.9)	34.6 (9.3)	0.30
EQ-5D, mean (SD)	0.66 (0.25)	0.70 (0.26)	0.30
SF-12 PCS, mean (SD)	38.1 (12.1)	41.2 (10.2)	**0.04**
SF-12 MCS, mean (SD)	48.0 (8.8)	47.8 (8.1)	0.86
Pain VAS, mean (SD)	64.5 (27.1)	70.6 (27.8)	0.14

*Unpaired *t* test

Bold numbers represent *p*-values of less than 0.1

*CR* cruciate retaining, *CS* condylar stabilizing, *EQ-5D* EuroQol-5 Dimensions score, *OKS* Oxford Knee Score, *PROM* Patient-reported functional outcome measure, *SD* standard deviation, *SF-12 MCS* Short Form-12 mental component summary score, *SF-12 PCS* Short Form-12 physical component summary score, *VAS* visual analogue scale

**Table 4:** Results of linear regression analysis to adjust for confounding variables (see Tables 1 and 2) between the two groups to assess the independent effect of group upon PROMs

PROM	Group	*B*	95% confidence interval		*p* value*
			Lower	Upper	
OKS	CR	Reference			
	CS	0.04	−2.78	2.85	0.98
EQ-5D	CR	Reference			
	CS	0.01	−0.08	0.09	0.90
SF-12 PCS	CR	Reference			
	CS	0.23	−2.99	3.46	0.89
SF-12 MCS	CR	Reference			
	CS	0.83	−1.59	3.26	0.50
Pain VAS	CR	Reference			
	CS	−5.20	−14.19	3.79	0.26

*Unpaired *t* test

*CR* cruciate retaining, *CS* condylar stabilizing, *EQ-5D* EuroQol 5 dimensions score, *OKS* Oxford Knee Score, *PROM* Patient-reported functional outcome measure, *SF-12 MCS* Short Form-12 mental component score, *SF-12 PCS* Short Form-12 physical component score, *VAS* visual analogue scale

The satisfaction rate was not significantly different between the CS group (*n* = 45, 83%) and the CR group (*n* = 304, 84%) (OR 0.94; 95%: CI 0.42 to 2.12; *p* = 0.56). Logistic regression analysis confirmed no significant difference in patient satisfaction according to group when adjusting for preoperative variables (OR 1.0; 95% CI 0.38 to 2.58; *p* = 0.99).

**Fig. 2:**
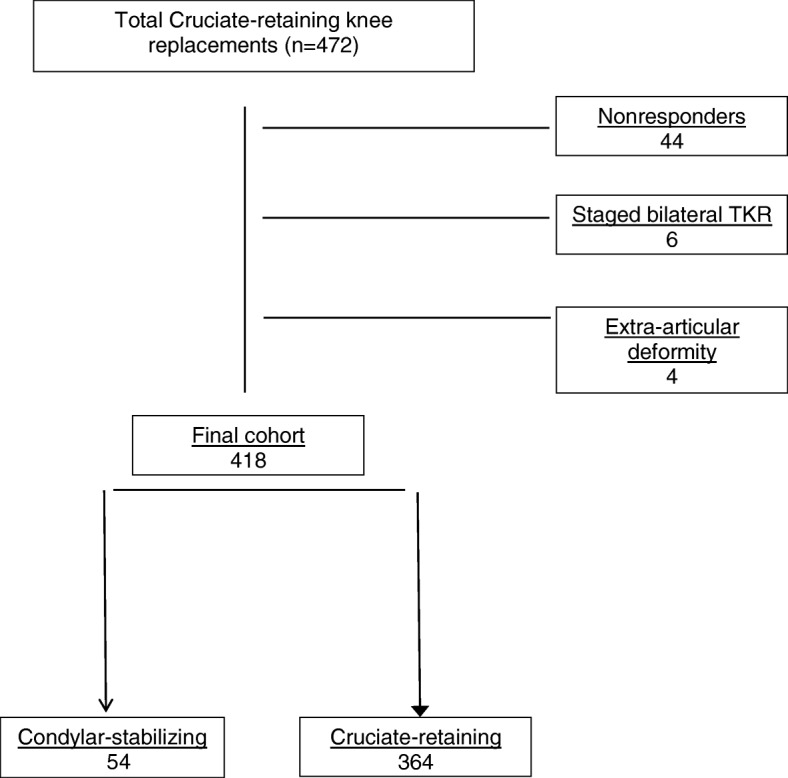
Schematic representation of patient enrolment. TKR total knee replacement

In total, 44 patients did not respond to their postoperative survey (9.5% lost to follow-up). Although this raises the possibility of non-response bias, the decision was taken not to contact these patients as they were past the 1-year postoperative stage and this would have increased the heterogeneity of our population. A schematic representation of enrolment in the study is outlined in Fig. 2.

## Discussion

This study has not been able to disprove the null hypothesis that there is no difference in early knee-specific functional outcome between the two groups. There was no significant difference in the knee-specific function (OKS as primary outcome measure), generic health measures (EQ-5D and SF-12), or patient satisfaction (secondary outcome measures) between those patients receiving a standard CR bearing surface and those receiving a more congruent CS bearing surface. Although a small difference of 3.1 points in the SF-12 PCS was noted between the groups, this was less than the previously reported minimum clinically important difference for this score [9], suggesting that this observed difference may not be clinically relevant.

There were similar patient demographics, early postoperative functional scores, and overall patient satisfaction following TKA with either a CS or a CR tibial bearing surface. The majority of studies investigating functional outcomes following TKA have focused on comparing CR and PS systems [10–15]. As a result, the different subcategories of CR TKA seem to have been overlooked in the literature and are frequently included together and compared en masse against non-CR designs. Berend et al. [4] reported a retrospective series investigating 2449 CR TKAs with three distinct tibial inserts: a standard insert with no posterior lip, a posterior lipped insert, and a highly congruent deep-dish insert designed for use with an attenuated or resected PCL. The main outcomes investigated were postoperative range of movement (ROM) and early reintervention with manipulation under anaesthesia (MUA) for an unacceptable ROM. Although this study was limited by a lack of PROMs, they report a significantly higher postoperative ROM, a lower rate of MUA, and no early revisions for instability in the 245 deep-dish knees when compared with the CR inserts with or without a posterior lip. Emerson et al. [16] reported a retrospective series of 930 TKAs performed using the Vanguard CR system (Biomet, Inc., Warsaw, IN, USA), of which 424 received a more congruent posterior lipped insert and the remainder received a standard insert with 3 degrees of posterior slope. They found no difference in survivorship or Knee Society functional scores between the two groups.

The common feature of deep-dish CS tibial insert design is an increased congruity of components through an arc of motion. Scott and Thornhill [17] first investigated this construct in 1994, finding no difference in postoperative ROM or tibial radiolucent lines between 50 knees with more congruent curved tibial inserts and 50 “standard” knees with no posterior lip. Hoffman et al. [18] also compared 100 CR TKAs with an ultra-congruent tibial insert with an age- and sex-matched control group of standard CR inserts using the Natural knee (Zimmer, Warsaw, IN, USA). They found no difference in functional outcomes but reported five cases of revision for anteroposterior instability in patients who received the standard CR insert compared with no revisions in the ultra-congruent deep dish group. Overall patient satisfaction following ultra-congruent tibial insert has been reported to be 92% at 2 years after surgery [19], and there are reports that flat tibial inserts may predispose to late instability due to excessive wear resulting from progressive tibiofemoral subluxation [20], although early wear has also been reported as a drawback of CS inserts [21]. More recently, ultra-congruent CS inserts have been shown to confer equivalent functional outcomes, anteroposterior stability, and postoperative ROM compared with CR inserts [22–24]. More congruent, deep-dish CS tibial inserts may be an attractive solution for surgeons aiming to maintain stability while avoiding the extra distal femoral bone resection required to use a PS implant.

This study shows no significant difference in early postoperative functional outcomes between CS polyethylene tibial inserts compared with standard inserts for primary CR TKAs. The main limitation of this study is that despite using prospectively compiled outcome data that are routinely collected at the study centre, the degree of PCL insufficiency and intraoperative kinematics were not recorded intraoperatively, and neither was the postoperative ROM. The postoperative ROM has been shown to correlate directly with the OKS [25], and in this study we relied upon patients’ subjective assessment of ROM as evaluated with the OKS rather than objective measurement in the clinic. A further limitation is the number of surgeons who performed the TKAs during the study period. Although this was a single-centre study, prejudices towards a CS or CR insert is a possible confounding variable. The decision to use a CS tibial insert was made by the operating surgeon at the time of component trialling based on several factors including soft tissue balancing and factors thought to influence stability such as BMI, inflammatory arthritis, or pattern of arthritis. It is possible that surgeons may be positively selecting patients who are predisposed to increased instability or postoperative complications for insertion of CS inserts, and this would make the comparison between the groups more problematic by introducing selection bias. The observed equivalent functional outcome and patient satisfaction scores would suggest that this is not the case.

## Conclusion

Congruent deep-dish CS tibial inserts have equivalent functional outcomes and patient satisfaction at 1 year after TKA when compared with standard CR tibial inserts. These inserts represent an option for surgeons undertaking CR TKA where increased congruency is desired. This study can only comment on early postoperative functional outcomes, and therefore longer term follow-up and a randomized controlled trial comparing both designs is suggested to determine whether CS inserts could be safely used in all primary knee replacements.

## Abbreviations

BMI: Body mass index
CR: Cruciate retaining
CS: Condylar stabilizing
DVT: Deep venous thrombosis
EQ-5D: EuroQol-5 Dimensions
MCS: Mental component summary
MUA: Manipulation under anaesthesia
OKS: Oxford Knee Score
PCL: Posterior cruciate ligament
PCS: Physical component summary
PROM: Patient-reported functional outcome measure
PS: Posterior stabilized
ROM: Range of motion
SF-12: Short Form-12
TKA: Total knee arthroplasty
